# The Tragus-Facial Angle: Proposition of a New Method to Estimate the Trajectory of the Frontal Branch of the Facial Nerve

**DOI:** 10.7759/cureus.101536

**Published:** 2026-01-14

**Authors:** Guilherme Gago, Antonio Strangio, Marc-Olivier Comeau, Yousef Odeibat, Martin Côte, Pierre-Olivier Champagne

**Affiliations:** 1 Department of Neurosurgery, Laval University, Quebec, CAN; 2 Department of Neurosurgery, Università di Torino, Turin, ITA; 3 Neurosurgical Innovation Laboratory, Laval University, Quebec, CAN

**Keywords:** facial nerve preservation, frontal branch of the facial nerve, orbitozygomatic approach, pterional craniotomy, skull base surgery, surface landmarks, tragus-facial angle

## Abstract

Introduction

Pterional craniotomy is a cornerstone of skull base surgery; however, extended approaches such as orbitozygomatic craniotomies require wider dissections, which increase the risk of injury to the frontal branch (FB) of the facial nerve. Several methods of varying complexity have been proposed to estimate the nerve’s trajectory, but no consensus has been established. This study aims to describe a new, straightforward method for estimating the FB position.

Methods

Cadaveric heads were dissected from the stylomastoid foramen to the distal part of the FB. The anterior edge of the tragus was set as the starting point A, point B was the vertical intersection with the facial nerve, and point C was the horizontal intersection with the FB. The angle between AB and BC was defined as the tragus-facial angle (TFA). Validation was performed against Pitanguy’s line using deviations between estimation methods and FB location.

Results

Eleven sides were dissected. The mean tragus-facial angle was 33.5° (range: 26.6°-37.7°). Key anatomical measurements included an average AB distance of 26.8 mm (SD ± 2.7), BC distance of 30.5 mm, AC distance of 16.9 mm, and A′-NF distance of 11.4 mm. The frontal branch trajectory followed the BC line, positioned approximately 2.5 cm below the tragus at an angle of ~30° to the vertical. Validation against Pitanguy’s classic line demonstrated no statistically significant differences across multiple measurement points (all *p* > 0.24), supporting the accuracy and reproducibility of the proposed method.

Conclusion

The tragus-facial angle method displayed acceptable variability and represents a simple alternative for estimating the position of the FB of the facial nerve during anterolateral approaches.

## Introduction

Pterional craniotomy is considered a cornerstone technique in skull base surgery, known for its versatility and broad applicability across various pathological conditions [1]. However, when employing this approach, particularly with inferior extensions of the skin incision for expanded approaches, such as orbitozygomatic craniotomies and their various modifications, surgeons encounter the challenge of performing more extensive soft tissue dissections. These wider dissections increase the risk of injury to the frontal branch of the facial nerve (FB), which can lead to significant postoperative complications, including aesthetic and functional deficits [2].

Injury to the FB is a well-known complication that can vary in severity, potentially resulting in either temporary or permanent paralysis. The incidence of FB paralysis ranges from 1.4% to 20.4% in frontotemporal approaches, with a notable association with orbitozygomatic craniotomies, where wide mobilization of the temporalis muscle is needed, but also wider dissection toward the zygomatic arch, which puts the surgeon closer to the FB [2-4].

A variety of techniques have been described to mobilize the temporalis muscle while preserving the FB, including interfascial dissection, subfascial dissection, and the mobilization of the temporalis muscle as a myocutaneous flap [5-9]. Regardless of the technique adopted, a thorough understanding of the trajectory of the facial nerve and its anatomical relationships with adjacent surface structures is crucial for preserving nerve integrity during these approaches. The frontal branch of the facial nerve typically follows a predictable path, but variations in its course are common, complicating intraoperative identification and safeguarding [10,11].

Numerous techniques have been proposed over the years to estimate the position of the facial nerve, ranging from simple anatomical landmarks to advanced imaging methods [12-14]. Additionally, monopolar stimulation has also been proposed as an auxiliary method [15,16]. However, despite the variety of approaches, a consensus remains elusive regarding the most effective and reliable techniques for intraoperative localization of this critical structure.

In this context, we aim to describe the tragus-facial angle (TFA) as a novel and straightforward method for estimating the position of the FB of the facial nerve during pterional craniotomy and its variations. We aim for this approach to assist in improving the consistency of facial nerve localization and contribute to reducing the risk of FB injury during cranial surgery.

This article was previously presented as a meeting abstract at the 2025 North American Skull Base Society (NASBS) Meeting on February 14, 2025.

## Materials and methods

The study was approved by the ethics committee of the Centre de Recherche du CHU de Québec-Université Laval (IRB #2025-7594). Eleven sides of formalin-fixed, silicon-injected cadaveric heads were dissected at Laval University Neurosurgical Innovation Laboratory (LUNIL). A ZEISS S8 Surgical Microscope (Oberkochen, Germany) attached to a high-definition camera and digital video recorder system was used together with a complete set of instruments for microsurgical dissections. Dissections were performed on 11 specimens from the stylomastoid foramen to the distal part of the FB of the facial nerve. The dissections were performed by two dissecting authors (AA and GG). Supervision was provided by the senior author (PC). Following each dissection, the anatomical measurements outlined below were collected to measure the TFA. Method validation was conducted on each specimen by comparing the deviation from the FB with a straight line derived from the TFA (TFA line) and Pitanguy’s line.

Tragus-facial angle method

**Figure 1:**
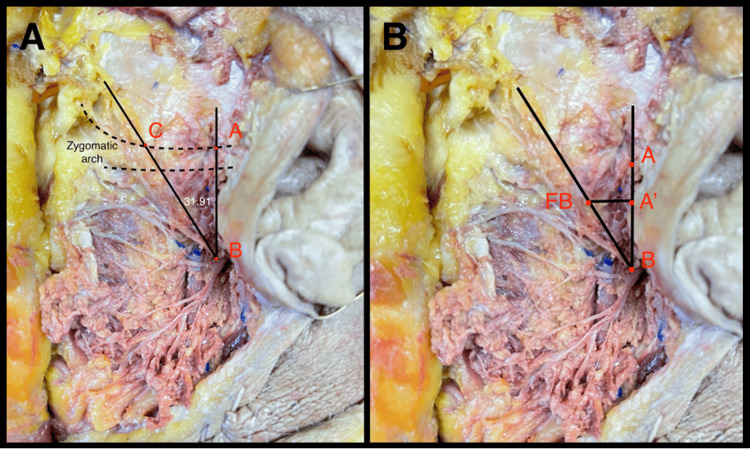
Tragus-facial angle calculation A: Anatomical dissection showing the method application. The angle between AB and BC (TFA line) was measured. In this specimen, the TFA was 31.91°. B: Demonstration of the method to estimate the position of the FB from the point A’ (point 1 cm below point A on the vertical plane) and the frontal branch horizontally. Point A (red): anterior edge of tragus, point B (red): vertical point of intersection between point A and the facial nerve, point C (red): horizontal point of intersection between point A and the FB of the facial nerve, point A’ (red): 1cm below point A, FB (red): frontal branch of the facial nerve TFA: tragus-facial angle, FB: frontal branch

The anterior edge of the tragus was defined as the starting point A, the vertical intersection of the anterior edge of the tragus with the facial nerve was set as point B, and the horizontal intersection of the anterior edge of the tragus with the FB was set as point C. Distances between points A, B, and C were measured. The angle between AB and BC (TFA line) lines was measured and defined as the TFA. We also measured the distance between A’ (point 1 cm below point A on the vertical plane) and the frontal branch horizontally (Figure 1B). The measurements were taken using a 15 cm surgical ruler (Medline, Northfield, IL).

**Figure 2:**
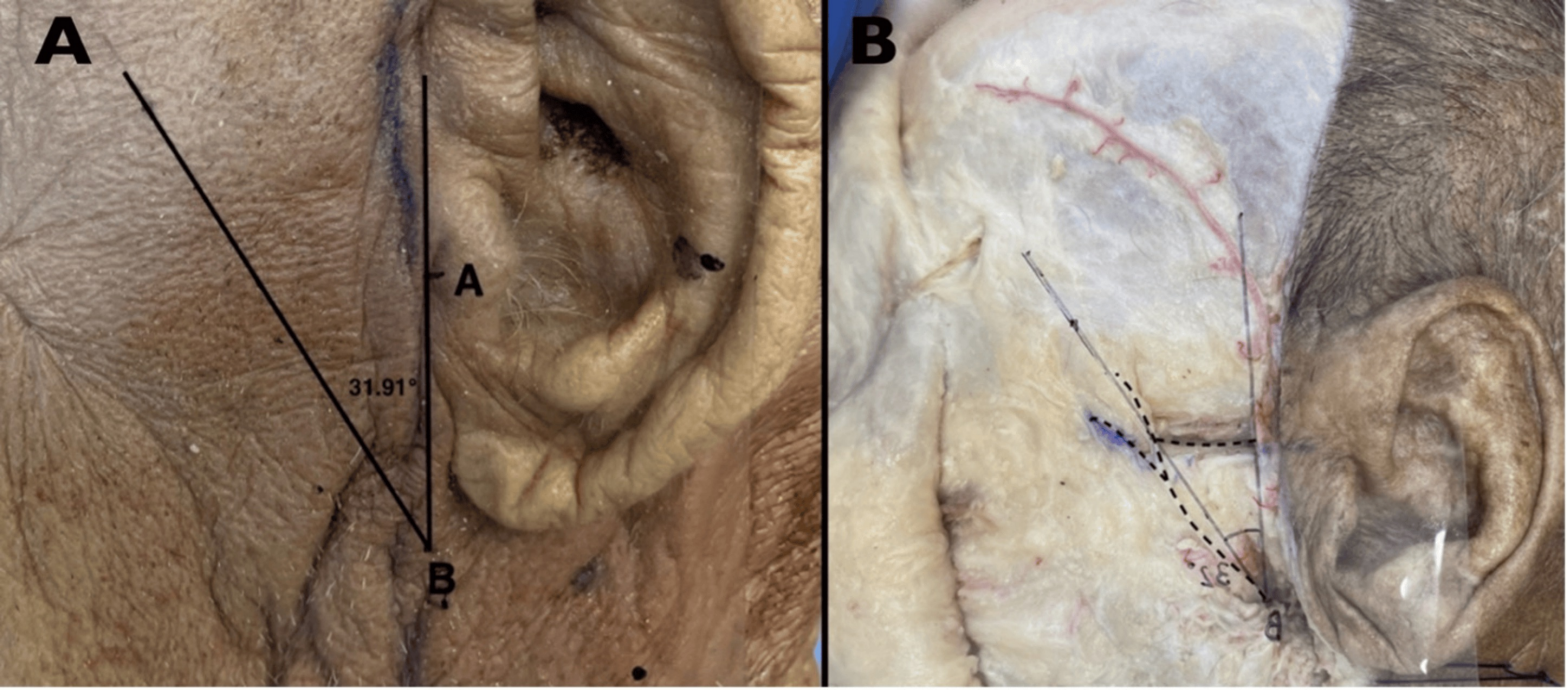
Application of tragus-facial angle A: Application of the TFA method on cadaver skin to estimate the position of FB. Facial nerve position could be estimated as the vertical distance from the anterior edge of the tragus, while FB position as the TFA line angled from the estimated facial nerve. B: Accuracy test of the method after dissection and exposure of the nerve’s trajectory. We drew the measurements on a transparent plastic sheet to assess the differences between the predicted position and the actual position of the FB. A horizontal dotted line indicates the superior border of the zygomatic arch. A curved vertical dotted line represents the course of the frontal branch of the facial nerve. TFA: tragus-facial angle, FB: frontal branch

We aim to propose the utilization of our measurements for the application of the TFA method to predict (1) facial nerve location based on vertical distance from the anterior edge of the tragus and (2) FB position based on the TFA line angled from the estimated facial nerve or horizontal distance from the anterior edge of the tragus (Figure 2).

Method validation

Method validation was conducted by comparing the deviation from the FB with a straight line derived from the TFA and Pitanguy’s line [13]. Pitanguy’s line is defined as a line drawn from a point 0.5 cm below the tragus of the ear to a point 1.5 cm above the lateral eyebrow. For direct comparison, the frontal branch (FB) of the facial nerve was exposed from its origin (point B, 0 cm) to the most distal segment visible during dissection. Along this trajectory, we marked the nerve at 1 cm intervals and measured the deviation (mm) between the true FB course and its predicted location according to Pitanguy’s line and the proposed TFA method.

Statistical analysis

Data are presented as mean ± standard deviation (SD). Overall deviation and deviation relative to the starting point were compared between the two methods using two-tailed Student’s t-tests. Maximal deviation was also assessed. Statistical analyses were performed using RStudio version 2023.12.1+402, and p-values < 0.05 were considered statistically significant.

## Results

**Figure 3:**
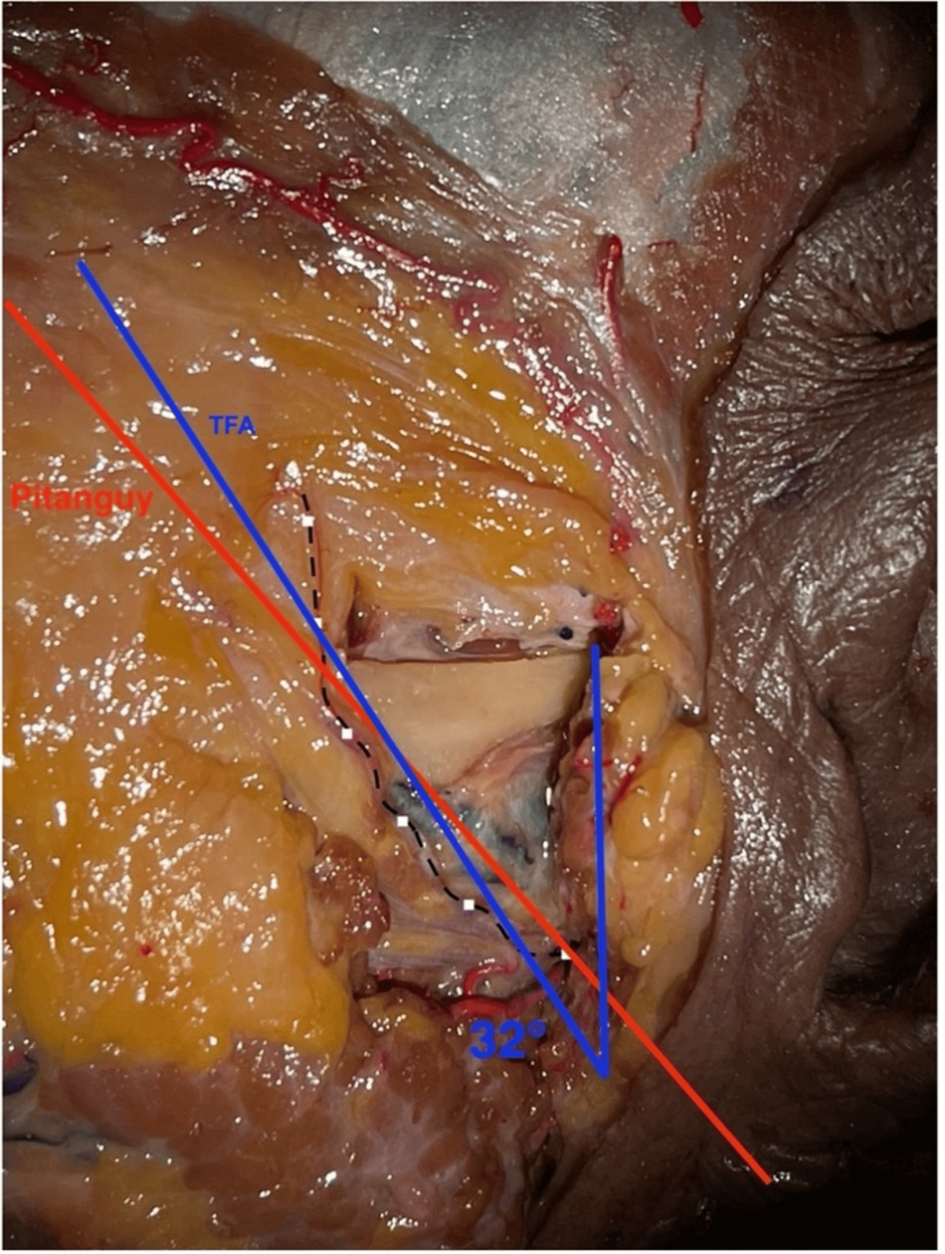
Demonstration of the tragus-facial angle and Pitanguy’s method on the same specimen Pitanguy’s method: red line *Pitanguy’s line is defined as a line drawn from a point 0.5 cm below the tragus of the ear to a point 1.5 cm above the lateral eyebrow. TFA line: blue line *The TFA line is defined as a straight line drawn from the estimated position of the facial nerve angled at the TFA. Based on our measurements, the facial nerve is positioned ~2.5 cm below the tragus, and the TFA was determined to be ~30°. Position of the frontal branch of the facial nerve: black dotted line

Eleven dissections were performed in six specimens and compared (Figure 3).

Anatomical measurements and tragus-facial angle

Anatomical measurements showed a mean AB distance of 26.8 mm (range: 20-29 mm), BC distance of 30.5 mm (range: 25-33 mm), AC distance of 16.9 mm (range: 13-19 mm), and A′-NF distance of 11.4 mm (range: 9-15 mm). The mean tragus-facial angle (ABC angle) was 33.5° (range: 26.6°-37.7°). Based on these data, the facial nerve aligns approximately 2.5 cm below the tragus, and the FB is positioned at a ~30° angle to the vertical.

Method validation

**Figure 4:**
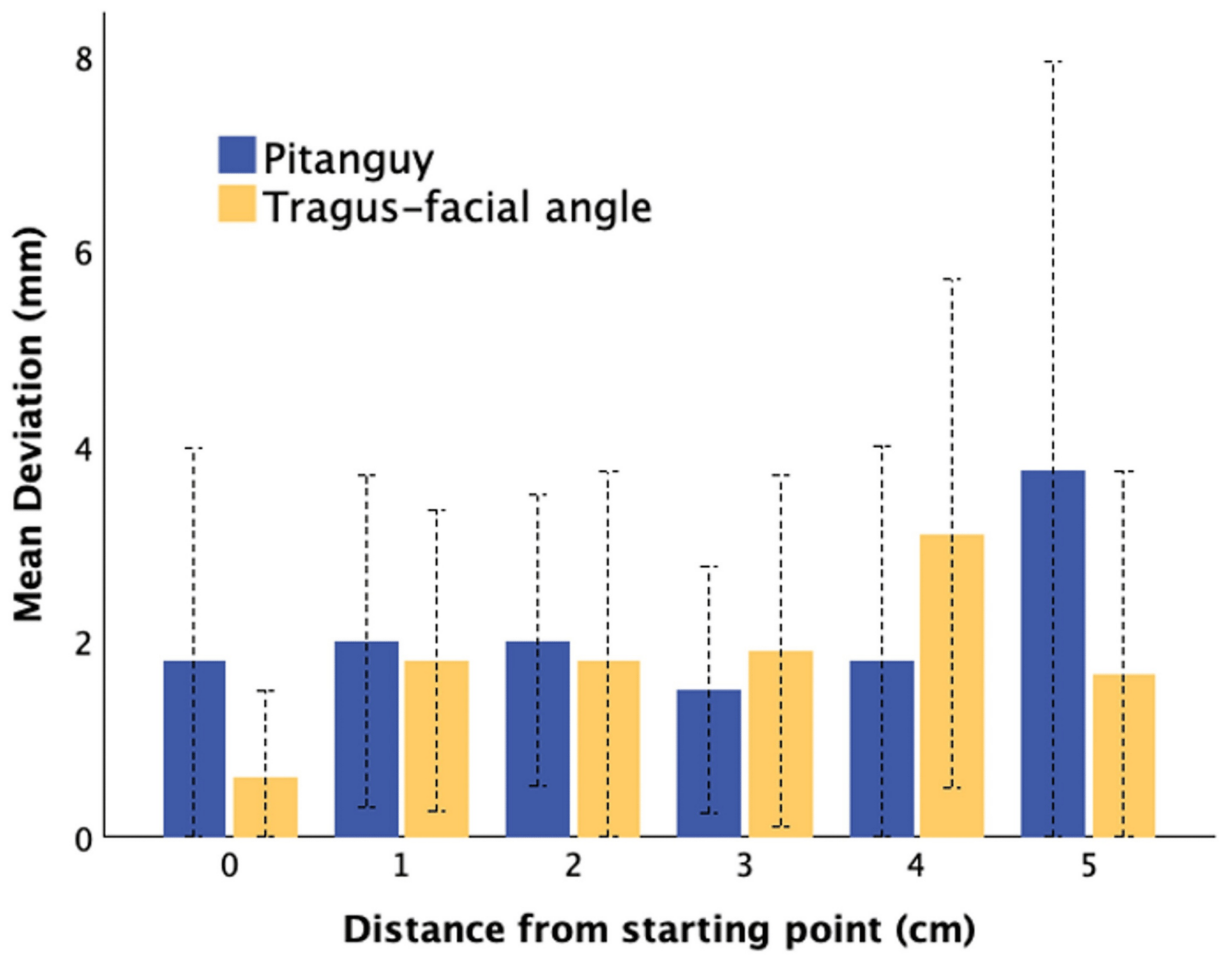
Mean deviation from FB according to both methods Mean deviation (mm) from the frontal branch of the facial nerve according to Pitanguy’s method (blue) and the proposed TFA method (yellow). Error bars represent one standard deviation. TFA: tragus-facial angle, FB: frontal branch

When comparing predictive accuracy, mean deviation from the actual FB was identical between Pitanguy’s method and the TFA method (2.0 ± 2.0 mm for both; p = 0.958). At each 1 cm interval from the nerve’s origin, deviations remained small (≤4.3 mm for Pitanguy and ≤3.1 mm for TFA) with no significant differences across all measurement points (all p > 0.24). Maximal deviation reached 10 mm for Pitanguy’s method and 8 mm for TFA. Detailed results are shown in Table 1 and illustrated in Figure 4.

**Table 1:** Comparison of mean deviation from FB Student’s t-test comparison of mean deviation from the frontal branch of the facial nerve according to Pitanguy’s method and the proposed TFA method. TFA: tragus-facial angle, FB: frontal branch

Distance from starting point	Pitanguy’s method mean deviation (mm)	TFA method mean deviation (mm)	p-value	t-statistic
0 cm	1.8 ± 2.2	0.6 ± 0.9	0.301	1.144
1 cm	2.0 ± 1.7	1.8 ± 1.6	0.786	0.275
2 cm	2.0 ± 1.5	1.8 ± 1.9	0.798	0.259
3 cm	1.5 ± 1.3	1.9 ± 1.8	0.572	-0.576
4 cm	1.8 ± 2.2	3.1 ± 2.6	0.243	-1.206
5 cm	3.8 ± 4.2	1.7 ± 2.1	0.472	0.778
Overall	2.0 ± 2.0	2.0 ± 2.0	0.958	0.053

## Discussion

In this study, we present a novel method, the tragus-facial angle, for estimating the position of the FB during frontotemporal approaches. This approach aims to define a safer surgical exposure zone to help reduce the risk of nerve injury.

The strength of this method is its simplicity: one only has to roughly draw a ~30° straight line (TFA line; FB position) from a point 2.5 cm below the tragus (facial nerve intersection) to estimate both FB and facial nerve position. Being more conservative with the angle and distance defines a safer exposure zone. Data from 11 specimens show reproducible narrow range facial nerve intersection (mean: 2.7 cm, range: 2.0-2.9 cm) and TFA (mean: 33.5°, range: 26.6°-37.7°). Our data suggest that anterior dissection extending beyond this angle may increase the risk of injury to the FB. Consequently, this method favors posteriorly positioned incisions and indicates that anterior extensions beyond 1 cm from the tragus should be approached with caution (the closest FB at that level was at 13 mm from the tragus).

We also observed that incisions placed up to 1 cm below the tragus remain within a safe zone for dissection, provided they are not extended too far anteriorly. In our samples, FB crossed the ZA between 13 and 19 mm anterior to the tragus along its superior border (AC) and was located between 9 and 15 mm anterior to a vertical line drawn 1 cm below the tragus. These measurements reinforce the spatial consistency of the FB’s course and support the use of surface landmarks to guide safe surgical exposure.

When compared with Pitanguy’s line, a long-standing anatomical reference for predicting the course of the frontal branch, the TFA demonstrated similar accuracy [13]. Our focused comparison showed that the mean absolute deviations between the two methods were comparable across most measurement points, and no statistically significant differences were observed. Rather than replacing Pitanguy’s line, the TFA may serve as a complementary and practical alternative, particularly in cases with greater anatomical variability or when more anterior dissection is needed. Its simplicity and reliance on reliable surface landmarks enhance its potential utility in surgical planning.

Historically, several techniques have sought to predict FB location using surface landmarks [17-19]. Lei et al. described a line from 0.5 cm below the tragus to 1.5 cm above the lateral eyebrow [17]. Correia and Zani proposed alternative topographies using the earlobe, forehead creases, or the canthus as reference points [19]. While helpful, these models offer limited precision in anterior extensions [17-19].

The TFA method complements previous surface landmark techniques by introducing an anatomy-based, angular measurement that demonstrated consistent accuracy in cadaveric dissections. Validation against Pitanguy’s classic line showed nearly identical predictive accuracy, with a mean deviation of approximately 2 mm and a maximal deviation of 8 mm from the frontal branch (FB) [13]. Our analysis also revealed that the FB consistently courses approximately 26.9 mm below the zygomatic arch when the incision is made vertically just anterior to the tragus, findings comparable to those of Gosain [20] and Perez-Rull et al. [21]. This narrow margin of error provides a reliable and reproducible safety buffer for clinical planning.

These results expand upon the conservative recommendations of Zabramski et al. [22] and Abdel Aziz [2], who advised limiting inferior skin incisions to about 10 mm below the zygomatic arch to reduce FB injury risk [9]. Our findings suggest that the FB may lie at a greater depth, around 27 mm below the arch, indicating a potentially wider safety margin than previously proposed. Nonetheless, adopting a conservative approach remains prudent, particularly in patients with anatomical variability or thick subcutaneous tissues, where estimating the nerve’s trajectory can be challenging. In this context, the concept of the “facial-zygomatic triangle” may be better understood through these quantifiable angular and linear relationships, potentially enhancing preoperative planning and intraoperative precision [12].

Overall, the introduction of the TFA may provide a useful surgical planning tool. It enhances anatomical understanding, offers a reproducible method using tangible surface landmarks, and is straightforward to implement. However, we emphasize that this technique, while anatomically validated, requires further in vivo validation to fully assess its safety and accuracy in clinical scenarios.

Limitations

This cadaveric study has several limitations that should be acknowledged. Firstly, the sample size was relatively small, which may limit the generalizability of the findings. Additionally, extrapolating the results to live patients must be done with caution, as tissue properties in living individuals could differ from those observed in cadaveric specimens. Furthermore, the measurements were obtained using an analog method, which may introduce potential inaccuracies due to manual measurement errors or minor variations in technique.

## Conclusions

The tragus-facial angle represents a simple, reproducible, and anatomically sound method for estimating the trajectory of the frontal branch of the facial nerve during pterional and orbitozygomatic craniotomies. When compared with the traditional Pitanguy’s line, the TFA demonstrates at least similar accuracy in cadaveric models and may provide added value in cases requiring anterior soft tissue dissections. Further clinical validation is warranted before broader adoption.
